# Prenatal Genetic Diagnosis in Three Fetuses With Left Heart Hypoplasia (LHH) From Three Unrelated Families

**DOI:** 10.3389/fcvm.2021.631374

**Published:** 2021-04-09

**Authors:** Sukun Luo, Luyi Chen, Weizhong Wei, Li Tan, Meng Zhang, Zhengrong Duan, Jiangxia Cao, Yan Zhou, Aifen Zhou, Xuelian He

**Affiliations:** ^1^Precision Medical Center, Tongji Medical College, Wuhan Children's Hospital (Wuhan Maternal and Child Healthcare Hospital), Huazhong University of Science and Technology, Wuhan, China; ^2^Prenatal Diagnosis Center, Tongji Medical College, Wuhan Children's Hospital (Wuhan Maternal and Child Healthcare Hospital), Huazhong University of Science and Technology, Wuhan, China; ^3^Ultrasonic Diagnosis Department, Tongji Medical College, Wuhan Children's Hospital (Wuhan Maternal and Child Healthcare Hospital), Huazhong University of Science and Technology, Wuhan, China

**Keywords:** prenatal diagnosis, congenital heart defects, *KMT2D*, *NOTCH1*, *WDFY3*

## Abstract

**Background:** Congenital heart defects (CHDs) are the most common birth defects, and left heart hypoplasia (LHH) is a severe form of CHD and responsible for more than 20% cardiac deaths during the first week of life, however, its genetic causes remain largely elusive.

**Methods:** Three families with fetal LHH were recruited. Genomic DNA from amniotic fluid or peripheral blood, and trio whole exome sequencing (trio-WES) and copy number variation sequencing (CNV-seq) were performed.

**Results:** All the three couples had no family history, and mid-gestation ultrasound revealed LHH and other variable cardiovascular defects in the fetuses. Trio-WES revealed *de novo* pathogenic variations in *KMT2D* (p.Gly3465Aspfs^*^37) (NM_003482) and *WDFY3* (p.Ser117Xfs^*^) (NM_014991), and CNV-seq identified a deletion of 150 kb encompassing *NOTCH1*. *KMT2D* and *NOTCH1* previously have been reported to be associated with CHDs, however, *WDFY3* is reported for the first time to be possibly related to CHD in human.

**Conclusion:** Our study suggested that genetic component is an important risk factor for the development of LHH, and next generation sequencing is a powerful tool for genetic diagnosis in fetuses with CHDs and genetic counseling, however, more studies and data are need to establish the correlation of fetal phenotypes and genotypes.

## Introduction

Congenital heart defects (CHDs) are a spectrum of diseases involving structural abnormalities of the heart and great vessels that affect about 1% of liveborn infants and occur in 10% of aborted fetuses (1). Left heart hypoplasia (LHH), a severe and complex phenotype of CHDs, refers to the underdeveloped left heart structures including left ventricle, aorta and mitral valve (2). Hypoplastic left heart syndrome (HLHS) is used to describe the most severe condition of LHH, and cause 23% of cardiac deaths in the first week of life without early surgery (3). Although a three-stage surgical palliation has significantly increased the 5-year survival rate (50–70%) (4), the extent of morbidity is considerable and life-expectance remains reduced, and some of them will still develop heart failure and need cardiac transplantation. To achieve substantive progress in the development of therapeutic intervention, the understanding of the formation of the 4-chambered vertebrate heart and the pathogenic mechanism causing LHH is needed.

Several lines of evidence support a genetic contribution to HLHS. A number of reports suggest HLHS is highly associated with chromosomal abnormalities (Turner, trisomy 13, 18, and 21) (5–7), as well as copy number variations (CNV) (DiGeorge, and Jacobsen syndrome) (8, 9). Genetic studies have also identified variants in several candidate genes such as *NKX2.5, GJA1, NOTCH1, HAND1, FOXC2, FOXL1, IRX4, MYH6*, and *RBFOX2*, in patients with HLHS (10–15), and these genes are very important for cardiogenesis.

The aim of this study is to identify the pathogenic variants in 3 fetuses with LHH by using next generation sequencing, and we found a *de novo* loss-of-function variant in *KMT2D*, and *WDFY3* (WD40 repeat and FYVE domain containing 3) gene, respectively, and a *de novo* heterozygous microdeletion variant in chromosome 9q34.3, encompassing *NOTCH1*, which aid prenatal genetic counseling and understanding of the pathogenesis of LHH.

## Methods

### Patient Enrollment

This study was approved by the Wuhan Children's Hospital (Wuhan Maternal and Child Healthcare Hospital) ethics committee and informed consent was obtained from the parents of the fetuses. We recruited 3 fetuses with LHH detected by ultrasound during middle gestation. The parents were healthy and non-consanguineous, and denied any adverse history. The maternal age range at diagnosis of LHH was 29–30 years, and the gestation age range at diagnosis was 23–25 weeks. Fetal samples were from amniotic fluid during the invasive prenatal diagnosis. A total of 2 ml of peripheral blood was collected in EDTA-containing tubes from the couples. Multidisciplinary consultation including genetic counseling regarding the risk of LHH and the chance of surgery as well as the benefits and limitations of whole exome sequencing (WES) and CNV sequencing (CNV-seq) were introduced to the couples.

### Sample Preparation

Genomic DNA was extracted from 20 ml of amniotic fluid or 2 ml whole blood according to the protocol of the Omega DNA Mini Kit. A NanoDropTM spectrophotometer was used for quality control (QC) of DNA purity and concentration.

### Whole Exome Sequencing and Data Analysis

Genomic DNA was sheared by sonication and exome sequences were enriched by IDT xGen® Exome Researcher Panel v1.0, according to the manufacturer's protocol. DNA libraries were sequenced on Illumina Hiseq XTen with a method of pair-end 150 bp reads. Raw image files were processed by the Bcl To Fastq (Illumina) for base calling and raw data production. Low quality variants were filtered out using the quality score ≥ 20 (Q20) standard. The sequencing reads were then mapped to the human reference genome (hg19/GRCh37) with BWA. Single nucleotide variations (SNVs) and Indels were scored by GATK 3.8. All short variants were annotated with databases including 1,000 genomes, dbSNP, Exac, GnomAD, ClinVar, HGMD, and OMIM. Impacts of variants on protein functions were predicted using software packages Provean, SIFT, Polyphen2_HDIV, Polyphen2_HVAR, Mutationtaster, M-CAP, and REVEL. The pathogenicity of candidate variants was evaluated on the base of mutated frequency, conservation of amino acid change and structural or function domain of the protein as well as inheritance pattern. The pathogenicity of identified variants was assessed in accordance with the American College of Medical Genetics (ACMG) guidelines and Sherloc (16, 17).

Generally, the variants detected by Trio-WES were analyzed according to ACMG standards and guidelines, and classified into pathogenic variants, likely pathogenic variants, uncertain significance variants, likely benign variants and benign variants. The former three types were further annotated in OMIM diseases database and filtered through family co-segregation according to genetic rules. Potential pathogenic genes were identified combining disease correlation and clinical observations. In addition, the incidental findings were disposed according to the document of ACMG about prenatal diagnosis (18). Pathogenic and likely pathogenic variants causing neurodevelopmental disorders, intellectual disability or metabolic diseases were reported.

### CNV Analysis

A total amount of more than 1.2 μg purified genomic DNA was broken into 200–300 bp fragments by sonication, followed by fragment DNA being sequentially repaired, tailed, ligated with an adaptor and amplified for library construction. The samples were then subjected to Illumina NovaSeq 6000 (Illumina, San Diego, USA). Raw data were processed by the basecall analysis software fastq v0.18.1. The clean data were then mapped to human reference genome (hg19) using BWA. The candidate CNVs were first filtered through normal frequency databases DGV, and, subsequently, were annotated based on scientific literature review and public databases including Decipher, ClinVar, ClinGen, ISCA, and dbVar. According to the ACMG, the candidate CNVs were classified into five categories: pathogenic, likely pathogenic, uncertain clinical significance, likely benign and benign (19).

### Sanger Sequencing

The identified variants in the *KMT2D* and *WDFY3* genes were confirmed by Sanger sequencing. Primers were designed to amplify the exon 36 of *KMT2D* and the exon 6 of *WDFY3*. PCR products were subjected to ABI 3730XL and analyzed by DNAStar software.

### Quantitative PCR Assay

Semi-quantitative qPCR was performed to confirm the presence of the microdeletion identified in case 3 by detecting the copies of DNA fragments within the region on both the fetus and the parents. Primers were designed to quantify the relative copies of *PMPCA* exon 2 and 13, *INPP5E* exon 10 as well as *NOTCH1* exon 1 and exon 34. The primers for these DNA fragments are listed as following: *PMPCA* exon 2(Forward: 5′-GTG CCT ATC CCA ACA TCC-3′ and Reverse: 5′-GCG AAG CCC ATT ATC CAA-3′), *PMPCA* exon 13(Forward: 5′-CCG TTC CCG TGC GTG TTA-3′ and Reverse: 5′-TTC CTG GCT TGC GGT GGT-3′), *INPP5E* exon 10(Forward: 5′-GCA CCA TCT GCT CCG TTT C-3′ and Reverse: 5′-TTC CTT CCT GGG ACG CTG-3′), *NOTCH1* exon 1(Forward: 5′-GAG CGC AGC GAA GGA ACG AG-3′ and Reverse: 5′-CCT CTC TTC CCC GGC TGG CT-3′) and *NOTCH1* exon 34 (Forward: 5′-GCA CAG GAG CGC ATG CAT CA-3′ and Reverse: 5′-GCT GGG CTT GCG GAC CTT CT-3′). The 36B4 gene was used as reference gene. q-PCR was performed on a ABI 7500 apparatus using SYBR Green Real-Time PCR Master Mix (Takara) according to the following conditions: 95°C for 2 min, followed by 40 cycles of 95°C for 15 s, 53°C for 15 s and 72°C for 34 s.

## Results

After performing genetic analysis, variants of two genes, *KMT2D* (NM_003482) and *WDFY3*(NM_014991), and a microdeletion involving *NOTCH1* were detected in three fetuses, respectively. All the three variants were classified into pathogenic variants because they were LOF and *de novo* mutations, and absent in control population. The detailed clinical features and genetic information were shown in Table 1. No other pathogenic variants in known pathogenic genes, which were in line with family separation, were identified in the three cases.

**Table 1:** Prenatal phenotype and genotype information for the cohort.

	**Case 1**	**Case 2**	**Case 3**
Maternal age	30 Y	29 Y	30 Y
Prenatal Gestational age	23 W	24 W + 3	25 W
Congenital heart defect	HLH, double-outlet right ventricle, perpetuate left superior vena cava, cardiac arrhythmias, single umbilical artery	Small left heart, coaractation of aortic arch, ascending arota, small VSD	HLH, atresia of mitral valves, stenosis of aortic valves, VSD
Extracardiac malformation	Horseshoe kidney	Congenital cystic adenomatoid malformation of the lung(type II), polydactylism	/
NT	3.5 mm	1.8 mm	1.2 mm
Karyotype	Normal	NA	Normal
CMA	Normal	del9q34.3	Normal
Pathogenic gene	*KMT2D*	*NOTCH1, INPP5E, PMPCA*	*WDFY3*
Variant	c.10394delG (exon36) NM_003482	/	c.349_c.350delAG (exon6) NM_014991
Amino acid change	p.Gly3465Aspfs*37	/	p.Ser117Xfs*1
AF in gnomAD	Absence	Absence	Absence
HGMD	Included	Not included	Not included
ClinVar	Not included	Not included	Not included
Zygosity	Heterozygous	Heterozygous	Heterozygous
Mutation type	Frameshift	Microdeletion	Frameshift
Parental origin	*De novo*	*De novo*	*De novo*
Disease	Kabuki syndrome 1	AOS 5/Aortic valve disease 1	Microcephaly 18
Inheritance Model	AD	AD	AD
Reference (PMID)	21671394	26820064	Novel
Pathogenicity	Pathogenic	Pathogenic	Pathogenic

^*^HLH, Hypoplasia left heart; VSD, ventricular septal defect; AOS, Adams-Oliver syndrome; AF, allele frequency; AD, autosomal dominant.

**Figure 1:**
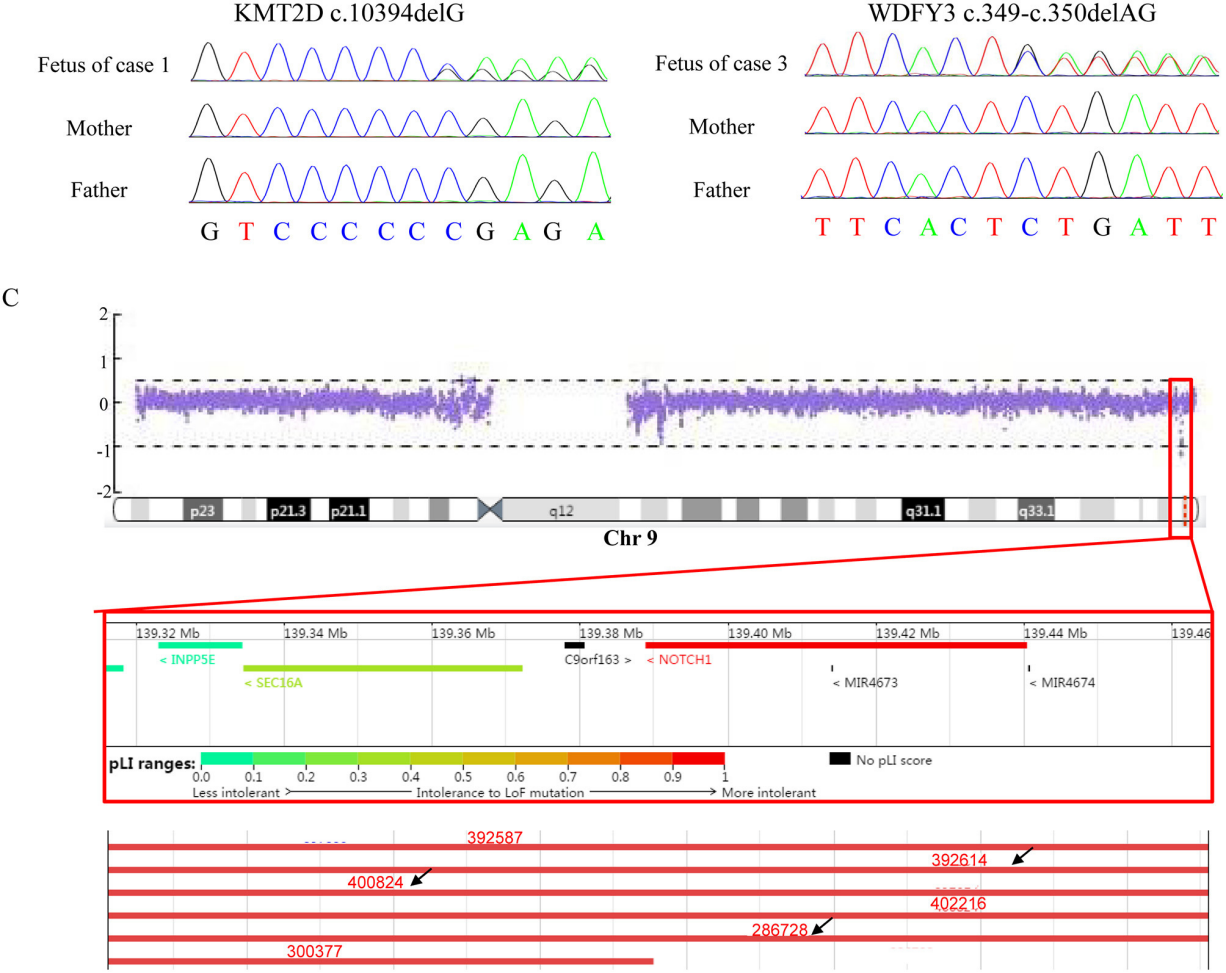
Results of Sanger sequencing and CNV-seq. **(A)** The mutation, c.10394delG, identified in *KMT2D* in the fetus (case 1) but not in the parents. **(B)** The mutation, c.394-c.350delAG, found in *WDFY3* in the fetus (case 3) but not in the parents. **(C)** A 150 kb microdeletion at 9q34.3 resulting in deletion of full-length of *NOTCH1* detected in case 2 and part of microdeletion variants encompassing the region in Decipher. Variants pointed by black arrow displayed heart abnormalities.

**Figure 2:**
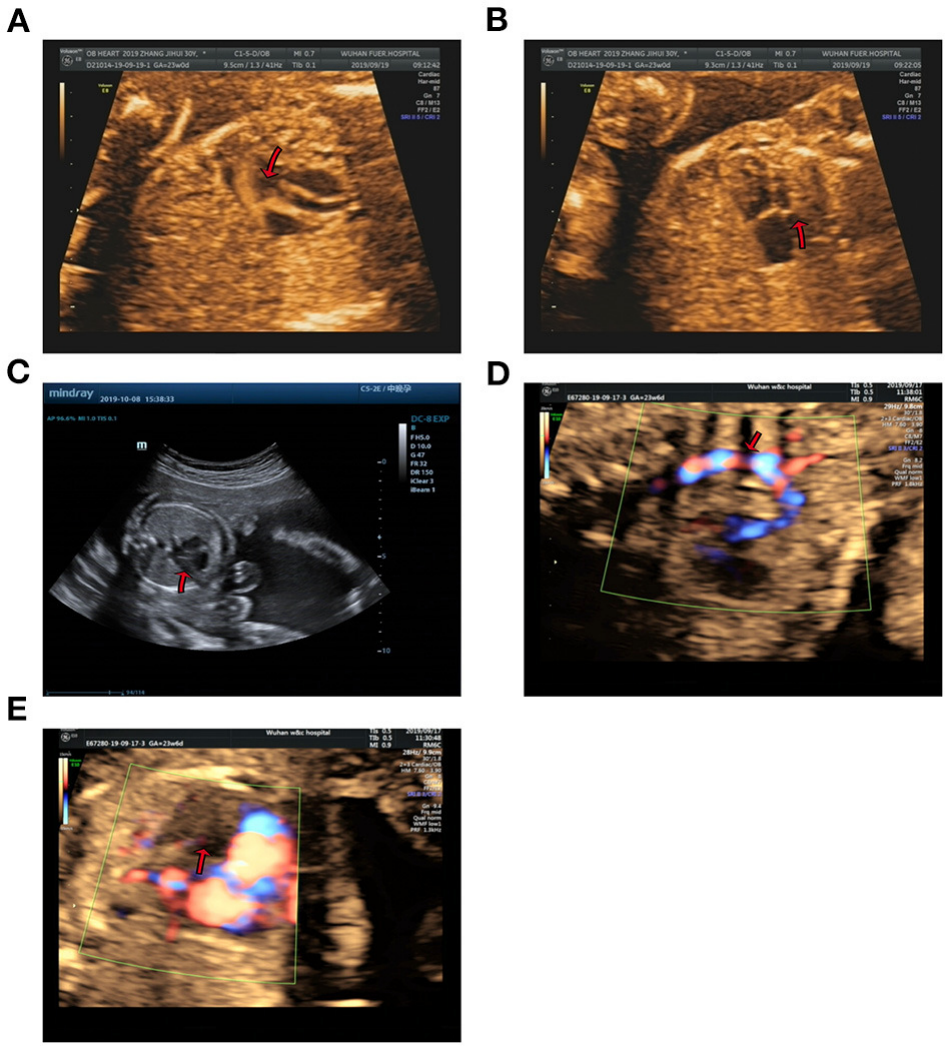
Echocardiographic images of fetal heart structures. **(A,B)** show fetal ventricular septal defect and hypoplasia of left heart in case 1. **(C)** displays a smaller left heart in case 2. **(D,E)** show coarctation of the aorta and mitral atresia in the fetal heart of case 3.

Case 1 was identified to be a *de novo* heterozygous variant (c.10394delG, p.Gly3465Aspfs^*^37) in the exon 36 of *KMT2D* gene (Figure 1A), which was reported previously and included in the HGMD (Accession number: CD114940) (20). At 23-week of gestation (wg), the ultrasound, shown in Figures 2A,B, revealed complex congenital heart defects (hypoplastic left heart, ventricular septal defect, double-outlet right ventricle, and perpetuate left superior vena cava), horseshoe kidney, and single umbilical artery, and the fetus presented arrhythmia. Mid-pregnancy ultrasound showed the fetus had increased nuchal translucency of 3.5 mm (normal value <2.5 mm) at 12 wg, and, at 18 wg, the tests of karyotyping and CMA analyses by amniocentesis were negative.

Case 2 was found to carry a *de novo* 150 kb microdeletion at 9q34.3 (chr9:139, 315, 643-139, 465, 759), resulting in deletion of full-length of *NOTCH1, INPP5E, and SEC16A* as well as part of *PMPCA* (Figure 1C). The ultrasound at 24 wg detected severe congenital heart defects, including small left heart (Figure 2C), coarctation of ascending aorta and aortic ache, and a small ventricular septal defect. Polydactyly and cystic adenomatoid of lung were also observed (Table 1). The nuchal translucency measurement and noninvasive prenatal testing were unremarkable.

Case 3 was identified to have a *de novo* heterozygous frameshift variant (c.349-c.350delAG, p.Ser117Xfs^*^1) in the exon 6 of *WDFY3* gene (Figure 1B), which was a novel pathogenic variant. Ultrasound of this fetus at 25 wg showed left heart dysplasia with mitral atresia and aortic valve stenosis (Figures 2D,E), and ventricular septum defect. No other abnormality was found during mid-pregnancy examination. Although, *WDFY3* pathogenic variants were reported to cause microcephaly or macrocephaly (21, 22), the described fetus in this case had normal head circumference.

## Discussion

Although increasing evidence supports a genetic etiology (23–25), the precise pathogenesis of LHH or HLHS is unknown. In this study, we reported 2 *de novo* frameshift variations in the *KMT2D* and *WDFY3* genes, respectively, and a 9q34.3 microdeletion of 150 kb encompassing *NOTCH1*, in three fetuses with LHH and other structural malformations.

**Figure 3:**
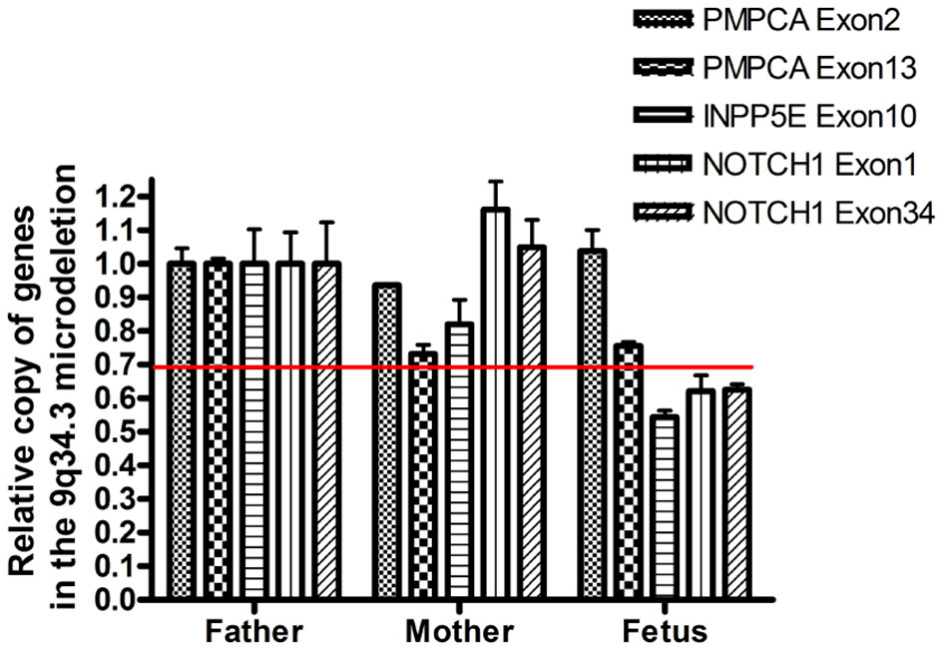
Confirmation of the presence of the deletion by semi-quantitative q-PCR. The copy number of *PCPMA, INPP5E, NOTCH1*, and *36B4* was evaluated by semi-quantitative PCR. The relative copies of *PCPMA* (exon 2 and 13), *INPP5E* (exon 10) and *NOTCH1* (exon 1 and 34) were normalized using, a valid single copy gene.

WES identified a *de novo* heterozygous frameshift variant (c.10394delG) in the *KMT2D* gene, resulting in a truncated protein, in Case 1. The *KMT2D* encodes a histone H3K4 methytransferases that is associated with transcriptionally active genes. Mutations in *KMT2D* are most frequently, up to 70%, found in patients with Kabuki syndrome (KS, MIM: #147920), which is a rare, autosomal dominant multiple congenital anomaly syndrome characterized by recognizable facial features, global developmental delay, intellectual disability, short stature, and musculoskeletal abnormalities (26). Approximately 55% of patients with *KMT2D* mutations have been reported to present CHDs, including both septal defects and aortic coarctation (27). Studies in animal models have indicated *KMT2D* is essential for regulating cardiac gene expression during heart development and is required for the establishment of the primary and secondary heart fields (28, 29). Recently, a study including 80 fetuses with congenital cardiac left-sided lesions showed that *KMT2D* was the most frequently mutated gene (10.6%) followed by *NOTCH1* (6.1%) (30). *NOTCH1*, encoding one of core receptors in Notch signaling pathway, is a well-known CHD-related gene, and loss-of-function variants in this gene confer a higher risk for and segregates with left-sided-CHD (31, 32). Deletion of the whole *NOTCH1* gene was previously reported to cause non-syndromic Tetralogy of Fallot and HLHS (32, 33). Our Case 2 carried a 9q34.3 microdeletion surrounding full-length *NOTCH1*, and presented underdeveloped left heart and ventricular septal defect. In addition to *NOTCH1*, the microdeletion contained three other protein-coding genes, *PMPCA, INPP5E*, and *SEC16A* according to human hg19/GRCh37. However, our q-PCR assay confirmed that the microdeletion might not destroy the *PMPCA* gene which contained 13 exons, since the relative copies of *PMPCA* exon2 and exon 13 in the fetus was comparable to that in its parents (Figure 3). *INPP5E* were associated with autosomal recessive diseases, while *SEC16A* had not been reported to be related to a known disease. Deletion of the whole *NOTCH1* gene was the most likely cause for LHH of the fetus in case 2.

Although a clear prenatal genetic diagnosis can be made only when definitely pathogenic genes and variants are identified, prenatal WES is invaluable for the diagnosis of causal genes but also for the discovery of candidate genes. In case 3, we did not detect any pathogenic variants in well-established CHD pathogenic genes, but identified a frameshift (c.349-c.350delAG, p.Ser117Xfs^*^1) variant in *WDFY3*, thus, we propose novel candidate gene for early cardiac development supported by functional and phenotyping evidence in animal model (34). *WDFY3* is located on chromosome 4q21.23, encompassing 74 exons and encoding a protein with 3,526 amino acids. The encoded protein is an adaptor protein involved in selective degradation of protein aggregates by autophagy, and is critical for brain development (35, 36). It was included in OMIM and associated with microcephaly 18, an autosomal dominant disorder. It is extremely intolerant to loss-of-function (LOF) mutation with a pLI score of 1. Pathogenic *WDFY3* variants caused mild to moderate neurodevelopmental delay and intellectual disability, most of which (8/13) were loss-of-function variant and putatively lead to haploinsufficiency (21).

Homozygous knockout of this gene in mice model would lead to variable cardiac anomalies, such as ventricular septum defect, double outlet right ventricle, aortic overriding, thinning of ventricular wall, ventricular dilation and disorganized trabeculation, and abnormal NOTCH1 signaling in the heart of mice (34), suggesting that *WDFY3* is critical for cardiac development. However, this report did not mention whether heterozygous *WDFY3* knockout mice presented visible heart abnormalities (34). Unfortunately, we did not obtain the heart tissue of the aborted fetus to examine the impacts of *WDFY3* variant on signaling pathways, such as NOTCH1 and Wnt pathways related to early cardiac development. This is the first report of a human phenotype caused by a frameshift variant in *WDFY3*, more cases and further functional studies are needed before we make a conclusion that *WDFY3* is pathogenic candidate gene for LHH. In this study, the pregnancy was terminated due to the poor prognosis of LHH and the risk of neurodevelopmental delay. The pregnant woman and her family were informed that the recurrence risk is very low except that the variant is moasic in germline cells.

In conclusion, next generation sequencing is a powerful tool for prenatal genetic diagnosis, in particular, for fetuses with LHH. Apart from two known genes, *KMT2D* and *NOTCH1*, we reported a potential pathogenic gene *WDFY3*, and this is the first case of a fetal CHD with a frameshift variant in *WDFY3*. Before this genetic variant is used to genetic counseling, more sequencing data and functional studies are needed to determine the contribution of this genetic variant to CHDs.

## Bulleted Statements

Genetic variants in *KMT2D* and *NOTCH1* previously have been reported in patients with CHDs, however, *WDFY3* is reported for the first time to be possibly related to CHD in human. Genetic factor is an important factor for the pathogenesis of LHH.

## Data Availability Statement

The data of KMT2D p.Gly3465Aspfs^*^ and WDFY3 p.Ser117Xfs^*^1 presented in the study are deposited in the ClinVar database, and the accession numbers were SCV001449147 and SCV001449148, respectively.

## Ethics Statement

The studies involving human participants were reviewed and approved by Wuhan Children's Hospital (Wuhan Maternal and Child Healthcare Hospital) ethics committee. The patients/participants provided their written informed consent to participate in this study.

## Author Contributions

HX and ZA conceived the study. CL, WW, ZM, DZ, CJ, and ZY clinically analyzed the patients. LS and TL conducted genetic data interpretation. LS and HX performed literature review and prepared the manuscript. ZA, ZY, and HX revised the manuscript. All authors reviewed and approved the manuscript.

## Conflict of Interest

The authors declare that the research was conducted in the absence of any commercial or financial relationships that could be construed as a potential conflict of interest.
